# Chimeric antigen receptor-engineered cell therapy for asthma: advances in targeting type 2 inflammation and translational challenges

**DOI:** 10.3389/fimmu.2026.1979396

**Published:** 2026-10-12

**Authors:** Yifei Niu, Chenyang Jian, Peize Liu, Mengwei Wu, Haoyuan Li, Xu Li, Lili Zhi

**Affiliations:** 1 Department of Respiratory, The First Affiliated Hospital of Shandong First Medical University, Shandong Provincial Qianfoshan Hospital, Shandong Institute of Respiratory Diseases, Jinan, China; 2 Department of Allergy, The First Affiliated Hospital of Shandong First Medical University, Shandong Provincial Qianfoshan Hospital, Shandong Institute of Respiratory Diseases, Jinan, China

**Keywords:** asthma, CAR-NK, CAR-NKT, CAR-T/CCAR-T, CAR-Treg, chimeric antigen receptor, type 2 inflammation

## Abstract

Asthma is a heterogeneous chronic airway disease, and some patients remain inadequately controlled despite optimized inhaled therapy and targeted biologics. Chimeric antigen receptor (CAR)-engineered cells are being explored as a programmable approach to depleting pathogenic immune cells, regulating local inflammation, and potentially achieving durable disease control. This narrative review evaluates the biological rationale, therapeutic targets, cellular platforms, engineering strategies, evidence levels, and translational challenges of CAR-engineered cell therapy for asthma. Available studies have directed CAR-based recognition toward IL-5Rα-positive eosinophils and their progenitors, membrane IgE-positive cells, FcϵRIα-positive effector cells, disease-relevant allergens, and tissue-associated antigens. However, no asthma-directed CAR product has undergone clinical validation. Clinical translation will depend on overcoming the limited disease specificity imposed by target heterogeneity and expression on normal immune populations, while ensuring that engineered cells remain sufficiently controllable in terms of persistence, reversibility, and toxicity. These biological challenges must also be balanced against the practical demands of conditioning, manufacturing, cost, and appropriate patient selection. Translation should prioritize human target validation, clinically relevant chronic disease models, controllable CAR designs, and carefully selected patients with severe, biologic-refractory type 2-high asthma. CAR-engineered cells represent a promising experimental platform rather than an established alternative to current asthma therapy.

## Introduction

Asthma is a heterogeneous disease comprising multiple clinical phenotypes and inflammatory endotypes (1, 2). In a substantial proportion of patients, type 2 airway inflammation is an important mechanism in disease pathogenesis and progression, commonly manifested by IgE-mediated allergic responses, eosinophilia, and activation of IL-4, IL-5, and IL-13 pathways (3–5). Most patients with mild-to-moderate type 2 inflammatory asthma respond well to inhaled corticosteroids (ICS) and bronchodilators (6). However, some patients with severe asthma and persistent type 2 features, including eosinophilic inflammation, remain poorly controlled despite guideline-directed high-dose ICS combined with LABA therapy (7, 8). Biologics directed against IgE, IL-5/IL-5Rα, IL-4Rα, or TSLP are now important treatment options for these patients (6, 9). Monoclonal antibodies reduce exacerbations and the need for systemic corticosteroids (10, 11). Current evidence, however, is insufficient to show that biologics modify the natural history of asthma, and clinical benefits generally wane after treatment discontinuation (12–14). Against this background, CAR-engineered cell therapy has been proposed as a new modality because of the capacity of these cells to persist *in vivo*, traffic to target sites, and undergo programmable modification (15–17). Whether these properties translate into durable clinical benefit and whether persistent CAR cells are safe in patients remain to be established in clinical studies (15, 16, 18).

CAR-engineered cell therapy first achieved major clinical success in hematologic malignancies and is now used to treat B-cell cancers, with applications in autoimmune disease also being explored (17, 19–21). The central principle is to genetically modify immune cells to express a synthetic receptor that specifically recognizes a target antigen (17). Unlike the TCR, a CAR generally recognizes cell-surface antigens without MHC presentation, providing a highly programmable platform for targeting defined pathogenic cells or tissue microenvironments (17).

To identify relevant literature, we conducted a structured narrative search of PubMed/MEDLINE, Embase, and Web of Science from database inception through 10 September 2026 using combinations of terms related to asthma, allergic airway inflammation, chimeric antigen receptor, CAR-T, CAR-Treg, CAR-NK, and CAR-NKT. We also manually screened the reference lists and citing articles of eligible studies and relevant reviews and searched conference-abstract archives for recent asthma-specific findings unavailable as full peer-reviewed reports. Primary experimental studies evaluating CAR-engineered cells in asthma or allergic airway models were classified as asthma-specific experimental evidence. Mechanistic and translational studies from other disease settings were included as extrapolated evidence when relevant to cellular platforms, engineering strategies, or safety. Conference abstracts were included only when they reported asthma-specific experimental findings and are identified as preliminary evidence throughout this review.

Existing reviews have mainly emphasized individual platforms or have surveyed CAR and chimeric autoantibody receptor approaches across broad hypersensitivity and autoimmune indications (22–24). In contrast, this review establishes an asthma-specific analytical framework to systematically evaluate chimeric antigen receptor-engineered cell therapies across multiple dimensions, including target recognition, cellular platforms, levels of evidence, and translational limitations. Based on this framework, this review first summarizes the major asthma-relevant targeting axes and their biological rationale, then compares the potential advantages, limitations, and applicable contexts of different cellular platforms, and further discusses advanced engineering strategies. On this basis, the review distinguishes direct evidence from extrapolated evidence, critically evaluates the existing studies, and further analyzes potential toxicities, risk-mitigation strategies, and key issues in clinical translation. Finally, this review discusses future research priorities and unresolved key questions and draws cautious conclusions based on the available evidence.

## Biological rationale for CAR-engineered cell therapy in asthma

Type 2-high asthma is closely associated with dysfunction of the airway epithelial barrier (3). Allergens, viruses, pollutants, fungi, and other environmental stimuli can damage or activate airway epithelial cells, inducing the release of epithelial alarmins such as TSLP, IL-25, and IL-33 and thereby activating dendritic cells, ILC2s, and TH2 cells (25–27). After antigen uptake and presentation, mature dendritic cells promote the differentiation of naive CD4+ T cells into TH2 cells in the presence of local cytokines and co-stimulatory signals (3, 5). TH2 cells and ILC2s amplify airway inflammation through the coordinated release of type 2 cytokines (3, 5). These cytokines interact to promote IgE responses, eosinophilic inflammation, mucus hypersecretion, and airway remodeling (4, 28). The epithelial alarmin-ILC2/TH2-type 2 cytokine network therefore provides a central immunologic framework for type 2-high asthma (25, 26). **Figure 1** illustrates the relationship between type 2 inflammatory pathways, candidate recognition targets, and the principal CAR-engineered cellular strategies discussed in this review.

**Figure 1:**
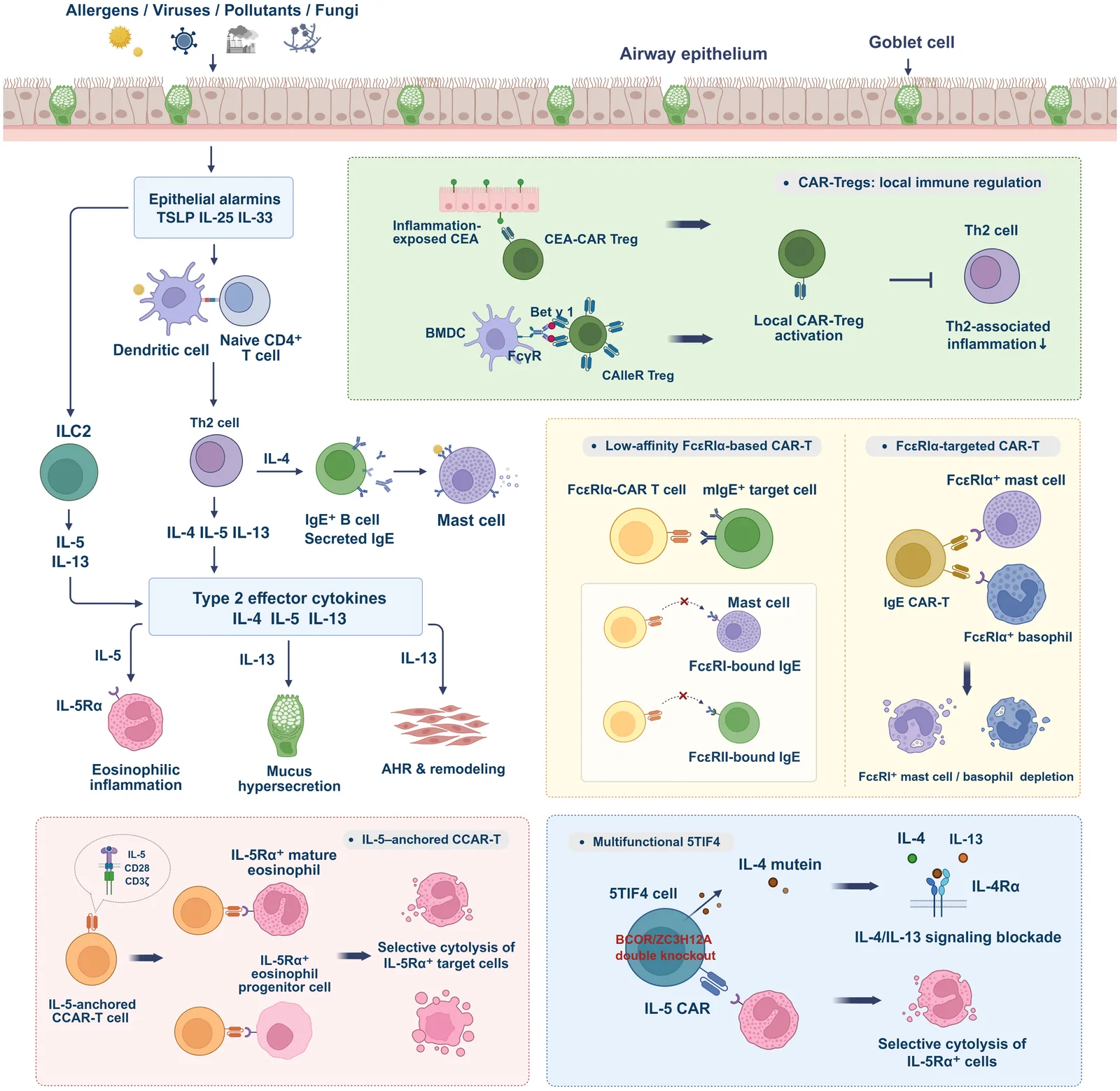
Type 2 inflammatory pathways in asthma and CAR-engineered cellular strategies. Epithelial alarmins activate ILC2 and TH2 responses, leading to IL-4-, IL-5-, and IL-13-mediated IgE production, eosinophilic inflammation, mucus hypersecretion, airway hyperresponsiveness, and remodeling. CEA-specific CAR-Treg and Bet v 1-specific CAlleR-Treg cells aim to suppress local allergic inflammation through antigen-dependent Treg activation. Two CAR-T strategies target different components of the IgE–FcϵRI axis: mIgE-targeted CAR-T cells eliminate mIgE-positive, IgE-producing cells, whereas FcϵRIα-targeted IgE-CAR-T cells eliminate FcϵRIα-expressing mast cells and basophils, with preliminary evidence for the latter from *in vitro* studies and an HDM-induced murine asthma model. IL-5-anchored CCAR-T cells deplete IL-5Rα-positive eosinophils and their progenitors. Multifunctional 5TIF4 cells combine IL-5Rα-positive cell depletion with secretion of an IL-4 mutein that blocks IL-4/IL-13 signaling. Evidence for the mIgE-targeted strategy is currently limited to *in vitro* studies; the CEA-CAR-Treg, CAlleR-Treg, IL-5-anchored CCAR-T, and 5TIF4 strategies have been evaluated mainly in mouse models, with no clinical validation in patients with asthma.

The intersection between asthma immunobiology and CAR technology lies in linking the recognition of disease-relevant molecular and cellular features to programmable therapeutic functions (15–17). Translating this concept into a therapeutic strategy therefore requires consideration of both the biological feature to be recognized and the effector system through which recognition is converted into a functional response.

## Therapeutic target landscape

### IL-5/IL-5Rα–eosinophil axis

Eosinophils are important effector cells in severe eosinophilic asthma (29, 30). By releasing granule proteins, lipid mediators, and cytokines, they can damage the airway epithelium, increase mucus production, and contribute to airway remodeling (29). The IL-5/IL-5Rα axis is central to eosinophil differentiation, maturation, and survival and underlies the efficacy of currently available biologics targeting IL-5 or IL-5Rα (4, 31). Unlike biologics, CAR-T strategies attempt to convert this inflammatory pathway into a depletion signal by engineering T cells to recognize IL-5Rα-positive cells and mediate cytotoxicity (16). A central question is whether sustained targeting of IL-5Rα-positive cells can achieve durable control of eosinophilic inflammation without unacceptable disruption of normal eosinophil-related functions (16, 29).

Chen et al. developed a scFv-independent, cytokine-anchored chimeric antigen receptor (CCAR) that used IL-5 as the extracellular target-binding domain (16). IL-5-anchored CCAR-T cells specifically targeted IL-5Rα-expressing eosinophils and eosinophil progenitors, selectively killed eosinophils *in vitro*, and inhibited progenitor differentiation. In mouse models of asthma, a single infusion of IL-5-anchored CCAR-T cells attenuated allergic airway inflammation and provided protection for approximately 3 months. The investigators also compared an anti-IL-5Rα scFv-based CAR, the IL-5-anchored CCAR, and CCR3-directed CAR designs. The IL-5-anchored construct showed greater *in vivo* activity than the comparator constructs under the experimental conditions examined (16). The study, however, relied mainly on prophylactic protocols in which cells were administered before allergen challenge; therapeutic effects in chronic asthma were not assessed, and systematic dose-response and long-term toxicity studies were lacking. Long-term persistence, effects on host defense, and safety in humans remain unknown (16). The 5TIF/5TIF4 strategy builds on the same IL-5/IL-5Rα recognition axis but incorporates additional engineering features that are discussed in the section on advanced CAR design (15, 16). A 2025 ERS Congress abstract also reported greater reductions in eosinophilic infiltration and pulmonary inflammation with IL-5R-directed CAR-T cells than with IL-13R- or FcϵRI-directed constructs in an OVA model, including protection after allergen rechallenge (32). However, the absence of a full peer-reviewed report, detailed quantitative results, and independent replication means that these findings should be regarded as preliminary (32).

### IgE-associated targets

IgE-mediated immunity is central to allergic asthma (30). Allergen-specific IgE sensitizes mast cells and basophils through FcϵRI (5). Subsequent allergen-induced cross-linking triggers mediator release, bronchoconstriction, mucus hypersecretion, and airway inflammation (5, 33). Omalizumab has validated the IgE–FcϵRI axis as a therapeutic target, but it primarily neutralizes circulating IgE and limits IgE–FcϵRI interactions rather than eliminating IgE-producing or IgE-responsive cells (4, 33).

Two CAR-T strategies have targeted opposite cellular nodes of this axis (33, 34). Ward et al. used the extracellular domain of FcϵRIα as a ligand-based recognition domain to target mIgE-positive, IgE-producing cells (33). This upstream strategy was intended to achieve durable suppression of IgE production while sparing FcϵRI-positive effector cells. Because soluble IgE could occupy the wild-type FcϵRIα domain and impair target recognition, affinity-reducing mutations were introduced to limit CAR blockade while preserving engagement with cell-bound mIgE. The resulting CARs retained activity against mIgE-positive targets in the presence of soluble IgE. They did not recognize IgE-loaded FcϵRI-positive mast cells or FcϵRII-positive B cells and did not induce mast-cell degranulation (33). By contrast, Sun et al. reported at the 2026 American Thoracic Society conference an FcϵRIα-directed CAR-T strategy designed to eliminate mast cells and basophils, thereby intervening downstream at the principal effector-cell level of IgE-mediated inflammation (34). According to the publicly available conference materials, the construct showed activity against FcϵRIα-expressing targets *in vitro* and was evaluated in an HDM-induced murine asthma model, in which reductions in basophils and eosinophilic inflammation were reported (34). These findings remain preliminary because a full peer-reviewed report with complete methods and quantitative data is not yet available. In particular, the consequences of engaging or lysing IgE-loaded FcϵRIα-positive cells, including acute mediator release and tissue mast-cell depletion, require careful evaluation (34). The two strategies are mechanistically distinct: mIgE targeting seeks upstream control by eliminating IgE-producing cells, whereas FcϵRIα targeting seeks downstream suppression through depletion of IgE-responsive effector cells (33, 34).

### Allergen-directed recognition

Allergen-directed recognition differs fundamentally from conventional CAR strategies targeting stable cell-surface antigens (17). Alcaraz-Serna et al. developed a chimeric allergen receptor (CAlleR) Treg strategy that directly targets a disease-relevant allergen (35). Variable regions from anti-Bet v 1 monoclonal antibodies isolated from B cells of birch pollen-allergic donors were formatted as scFvs and used as the extracellular recognition domains of CAlleR-Treg cells. Bet v 1 was selected because of its clinical relevance to birch pollen allergy and its homology with other PR-10 allergens (35). The feasibility of this strategy also depended on how the soluble allergen was presented. Monomeric Bet v 1 did not induce sufficient CAR clustering for effective activation. Productive signaling required a noncompeting anti-Bet v 1 IgG to anchor the allergen to FcγR-positive antigen-presenting cells while preserving the CAlleR-binding epitope (35). This mechanism extends CAR recognition beyond constitutively expressed surface antigens. It also demonstrates that pathogenic relevance alone is insufficient for selecting a soluble target. Antigen valency, epitope geometry, endogenous antibody abundance, and FcγR availability become integral design variables. This dependence represents an important translational constraint. In the limited donor samples examined, anti-Bet v 1 IgG levels were lower than those in sensitized mice, and the sera did not support CAlleR8 activation *in vitro (*35). Combinations of noncompeting recognition domains or exogenous bridging antibodies may reduce this dependence, although these approaches would increase therapeutic complexity and require independent safety evaluation. Thus, allergen-directed chimeric receptors establish allergen-triggered engineered-cell activation as a distinct design principle. Their generalizability depends on whether each allergen can be presented in a configuration that supports receptor clustering and elicits the intended function in the selected cellular platform (35).

### Tissue-directed recognition

Skuljec et al. generated CEA-specific CAR-Treg cells and evaluated them in an OVA-induced model of allergic airway inflammation in human CEA-transgenic (CEAtg) mice (36). The authors proposed that inflammation-induced epithelial disruption exposes CEA on the stromal side, where it becomes accessible to systemically delivered CAR-Tregs. Thus, a tissue-associated self-antigen that does not drive asthma was repurposed as a local trigger for immunoregulation. CEA-specific CAR-Tregs accumulated in the lungs and tracheobronchial lymph nodes and were more effective than unmodified Tregs in reducing airway hyperresponsiveness, eosinophilic inflammation, mucus production, and type 2 immune responses. Their loss of relative advantage in CEA-negative mice further supported a role for antigen-dependent CAR activation. However, the proposed inflammation-dependent exposure of stromal CEA was not directly quantified, and CAR-Tregs also accumulated in the spleen and stomach. CEA therefore did not function as a lung-exclusive address. CAR-associated tonic signaling and sustained recognition of an endogenous antigen may also complicate control of Treg activation and phenotypic stability (36, 37). These limitations indicate that the transferable concept is tissue-directed regulation rather than CEA itself (36). Candidate targets should be selected according to disease-dependent accessibility, restricted off-tissue expression, and the capacity to retain regulatory cells without persistent systemic activation (36, 37). Target selection must also remain aligned with the cellular platform because a self-antigen suitable for CAR-Treg localization could be unsafe when coupled to a cytotoxic effector cell (17, 36).

## Cellular platforms and therapeutic functions

Once a recognition target has been selected, the cellular platform determines how antigen engagement is translated into cytotoxic, regulatory, or other therapeutic functions (17). Platform selection should therefore reflect the intended therapeutic output, required duration of activity, and acceptable safety profile.

### CAR-T

Conventional T cells currently provide the principal experimental platform for cytotoxic CAR strategies in asthma. CAR engagement converts target recognition into cytotoxicity, clonal expansion, and potential memory formation (17). These properties provide a rationale for evaluating CAR-T cells for sustained depletion of pathogenic immune cells and their progenitors (15, 16). They also explain why the IL-5/IL-5Rα and mIgE recognition strategies described above were initially evaluated in T-cell-based systems (16, 33). The 5TIF4 platform further demonstrates that T cells can accommodate additional modifications that enhance persistence or deliver therapeutic proteins (15, 38). However, the available studies establish only preclinical feasibility and do not demonstrate that conventional T cells are superior to alternative platforms in asthma (15, 16, 36). Their strong expansion and prolonged persistence may extend the depletion of normal target-expressing cells and make unintended activity difficult to reverse (17, 39). Potential lymphodepleting conditioning and systemic immune toxicity further narrow the acceptable therapeutic window in a chronic nonmalignant disease (40–42). Conventional CAR-T cells may therefore be most appropriate when durable target-cell depletion is required, the physiological consequences of that depletion are acceptable, and effective termination mechanisms are available (40, 43).

### CAR-Treg

Treg cells are essential for maintaining peripheral immune tolerance and limiting excessive inflammation (44, 45). They suppress allergic immune responses through IL-10 and TGF-β secretion, attenuation of co-stimulation by antigen-presenting cells, and restriction of effector T-cell proliferation (44, 46). Enhancing or supplementing Treg cell function has therefore been considered a potential strategy for controlling allergic inflammation (36, 46).

Conventional Treg cell therapy nevertheless has important limitations (44). Ex vivo-expanded polyclonal Treg cells have diverse and uncertain TCR specificities, limiting their enrichment at sites of inflammation (44, 45, 47). Without local antigen recognition and sustained activation signals, their survival, expansion, and suppressive function in diseased tissues may be inadequate (44, 45). Expression of a CAR enables Treg cells to recognize a defined tissue antigen in an MHC-independent manner (47). CAR-mediated antigen recognition does not itself confer tissue-homing capacity, but it can promote local retention, activation, and functional maintenance at sites expressing the target antigen (36, 47). Compared with polyclonal Treg cells, CAR-Treg cells could concentrate suppressive activity within diseased tissue, improving local immune regulation while limiting systemic nonspecific immunosuppression (36, 44, 47).

### CAR-NK

CAR-T cells can be potent and persistent, but the acceptable threshold for treatment-related toxicity is substantially lower in chronic nonmalignant diseases such as asthma than in cancer (23, 40). NK cells are early immune responders that can mediate cytotoxicity without classical HLA-restricted antigen presentation (48). After CAR engineering, NK cells retain both CAR-mediated antigen specificity and native recognition through endogenous receptors (49). This dual recognition could broaden target-cell recognition and may improve activity within the complex inflammatory environment of asthma (49). A proposed advantage of CAR-NK cells over CAR-T cells is a lower risk of severe toxicities (48, 50). Their relatively limited *in vivo* lifespan may contribute to lower risks of CRS, ICANS, and GVHD, although shorter persistence could also compromise long-term control of chronic inflammation (49, 50). CAR-NK products can be generated from peripheral blood, umbilical cord blood, NK cell lines, or iPSCs, supporting standardized manufacturing and off-the-shelf use (48, 51). No direct preclinical or clinical evidence currently supports CAR-NK therapy for asthma (23). The rationale is extrapolated from CAR-NK studies in cancer, immunologic studies of NK cells in asthma, and existing CAR-T and CAR-Treg studies in asthma (23, 48). CAR-NK cells should therefore be viewed as a candidate platform rather than an established asthma therapy (23).

### CAR-NKT

Natural killer T (NKT) cells comprise heterogeneous subsets of innate-like T lymphocytes. Invariant NKT (iNKT) cells express a semi-invariant TCR that recognizes CD1d-presented lipid antigens and can rapidly mediate cytotoxicity and cytokine release (52, 53). The role of iNKT cells in asthma is context dependent (52). Some iNKT cells promote eosinophilic inflammation and airway hyperresponsiveness through IL-4, IL-5, and IL-13, whereas IFN-γ and IL-10 produced under particular stimuli, differentiation states, or disease stages may suppress TH2 responses (22, 52). NKT cells are therefore not intrinsically anti-inflammatory; subset composition, activation state, and cytokine bias are likely to shape the efficacy and safety of CAR-NKT cells (22, 52). These properties provide a rationale for CAR-NKT development in asthma but also call for careful functional engineering (22).

Several candidate CAR-NKT configurations have been proposed for asthma, although none has yet been evaluated in asthma-specific preclinical models (22, 54). Recognition modules could potentially be adapted from strategies explored with other CAR platforms, including FcϵRIα-based recognition of mIgE-positive B cells or IL-5-based recognition of IL-5Rα-positive eosinophils and their progenitors (16, 33). CAR-NKT function could also be tuned through costimulatory or immunoregulatory modules, such as 4-1BB, ICOS, CTLA-4, and PD-1/PD-L2, to modulate activation, persistence, and cytokine output (53, 55). These approaches remain conceptual and require experimental validation in asthma. Future studies should define the appropriate NKT phenotype and determine whether the intrinsic CD1d-dependent recognition and immunoregulatory properties of NKT cells provide advantages that can be meaningfully exploited after CAR engineering (53).

## Advanced engineering strategies

### Long-lived and multifunctional 5TIF4

The IL-5-anchored CCAR-T study established the feasibility of targeting eosinophils in asthma, whereas the 5TIF/5TIF4 strategy extended this concept to long-term cellular persistence and multi-pathway intervention (15, 16). Building on the same IL-5-IL-5Rα recognition principle used by Chen et al., Jin et al. generated baseline IL-5 CART cells, termed 5T cells. Although these cells specifically lysed IL-5Rα-positive cells *in vitro*, they failed to expand effectively or deplete eosinophils in immunocompetent mice that had not received lymphodepleting conditioning. Combined deletion of BCOR and ZC3H12A generated 5TIF cells that expanded without conditioning, persisted *in vivo* for at least 1 year, and maintained long-term depletion of IL-5Rα-positive eosinophils (15).

The investigators subsequently engineered 5TIF4 cells to constitutively secrete an IL-4 mutein. This mutein antagonized IL-4Rα and blocked both IL-4 and IL-13 signaling (15). Compared with 5TIF cells, which acted primarily through eosinophil depletion, 5TIF4 cells more strongly attenuated the positive-feedback circuitry of type 2 inflammation and the reactivation of memory TH2 cells, with secondary reductions in IL-5 and IL-13. They also suppressed plasma-cell responses and antibody class switching, reducing total IgE and allergen-specific IgE and IgG1. 5TIF4 cells established residence in nonlymphoid tissues, including the lungs, where they could continue to survey target cells and release the IL-4 mutein. Across acute, chronic, established, and relapsing models induced by ovalbumin (OVA), house dust mite (HDM), or IL-33, a single 5TIF4 infusion without conditioning durably suppressed pulmonary inflammation and alleviated asthma-like manifestations (15). The construct was also reproduced in human T cells, providing preliminary evidence that the engineering strategy can be implemented in a human cellular system. Although 5TIF4 combines target-cell depletion with delivery of an anti-inflammatory protein in a single cell, secretion of the mutein has not been shown to be strictly coupled to CAR antigen recognition. Conditional secretion circuits triggered by target antigen or inflammatory signals might provide a more favorable balance between local efficacy and systemic safety than continuous constitutive secretion.

In mouse models, this study showed that multifunctional CAR-T cells may act on several pathological components of asthma, supporting further evaluation of modular designs. The available data do not distinguish whether durable control resulted primarily from sustained eosinophil depletion or from continuous local release of the IL-4 mutein. Future studies could separately define the contribution of each function by using designs that permit inducible termination of CAR-mediated cytotoxicity or cessation of IL-4 mutein secretion (15, 43, 56). Efficacy and safety also require assessment in models that more closely approximate clinical disease and ultimately in human studies (57, 58). The long-term safety of BCOR and ZC3H12A editing, together with the limited controllability of cells engineered for prolonged and potentially irreversible persistence, must be resolved before clinical translation (15, 43, 59).

### Advanced CAR architectures for precision and control

Asthma has marked endotypic heterogeneity, and a single target may address only part of its pathogenic network (2, 9). Dual- or multi-target CARs can recognize different pathogenic cells or molecules, whereas logic-gated CARs can determine cellular activation from defined antigen combinations; both approaches may improve target specificity (60–62). Their feasibility depends on identifying antigen combinations with stable co-expression patterns that distinguish diseased from normal tissues (39). This condition may be difficult to meet because major asthma pathways are distributed across eosinophils, B cells, mast cells, and airway structural cells (3, 4). Multifunctional engineering may therefore represent an alternative to conventional antigen-logic approaches in some endotypes, although this possibility has not been directly tested (56, 63). 5TIF4 cells can be viewed as an early example of an armored, multifunctional CAR-T cell design for asthma (15, 56). Because multifunctionality and prolonged persistence may also broaden therapeutic risk, such strategies will require designs that preserve reversibility and controllability; these safety considerations are discussed in detail below (40, 43, 56).

## Comparative evidence, heterogeneity, and unresolved questions

Despite substantial methodological heterogeneity, several areas of convergence are evident. Across the available asthma-directed studies, CAR-mediated recognition can be redirected toward distinct components of type 2 inflammation, and antigen-dependent activation has produced measurable suppression of allergic inflammation in several murine models (15, 16, 36). The studies also consistently indicate that *in vivo* efficacy depends on more than receptor binding or cytotoxicity observed *in vitro*, with antigen accessibility, cellular persistence, and the inflammatory context emerging as important determinants of therapeutic activity (15, 16, 35).

The clearest cross-study comparison concerns IL-5/IL-5Rα-directed cytotoxic T-cell strategies. Chen et al. and Jin et al. both support the feasibility of sustained depletion of IL-5Rα-positive cells *in vivo (*15, 16). However, important differences are evident. In the original CCAR study, the IL-5-anchored receptor and an anti-IL-5Rα scFv-based CAR showed similar target recognition and effector activity *in vitro*, whereas only the IL-5-anchored construct reduced eosinophilia *in vivo*. CCR3-directed ligand receptors were also ineffective. These findings suggest that *in vitro* potency does not necessarily predict activity in an intact host (16). A second apparent discrepancy is seen between the prolonged protection reported with unedited IL-5-anchored CCAR-T cells in allergen-challenged mice and the limited expansion of baseline 5T cells in nonconditioned mice reported by Jin et al (15, 16). This difference should not be interpreted as a direct conflict because the studies differed in mouse strain, disease state, antigen burden, T-cell composition, dose, and assessment time. The CAR-Treg studies provide a second example in which differences in target biology may influence the observed therapeutic effect (35, 36). Both CEA-specific CAR-Treg cells and Bet v 1-specific CAlleR-Treg cells support the principle that antigen recognition can enhance regulatory activity in allergic airway inflammation. However, the mode of antigen presentation differs substantially between the two strategies. CEA is a cell-associated tissue antigen, whereas Bet v 1 is a soluble allergen that requires FcγR-mediated anchoring through a noncompeting IgG to support effective CAlleR clustering and activation. Differences in the observed activity of these approaches may therefore reflect antigen accessibility and presentation rather than intrinsic differences in the regulatory-cell platform. Studies targeting mIgE and FcϵRIα should not be presented as conflicting evidence (33, 34). They intervene at different points in the IgE axis and have been evaluated at different evidence levels. Available evidence includes *in vitro* proof of recognition and specificity for mIgE targeting and preliminary conference-reported *in vivo* findings for FcϵRIα targeting. No peer-reviewed matched comparison between these strategies or clinical study in asthma is available (33, 34). Human efficacy, comparative target value, long-term safety, and disease modification therefore remain unresolved (24). CAR-NK, CAR-NKT, and most logic-gated designs remain hypothesis-generating concepts (22, 23, 63). The representative asthma-directed and extrapolated studies discussed in this review, together with their experimental models, principal findings, limitations, and levels of evidence, are summarized in **Table 1**.

**Table 1:** Representative studies and levels of evidence for CAR-engineered cells in asthma and allergic airway inflammation.

Study	Cell/platform	Target	Experimental model	Evidence level	Principal findings	Limitations
Skuljec et al., 2017 (36)	CAR-Treg	Airway epithelial CEA	OVA-induced allergic airway inflammation in CEA-transgenic mice	*In vitro* + *in vivo* preclinical	Reduced AHR, eosinophilic inflammation, mucus production, TH2 cytokines, and OVA-specific IgE.	Inflammation-dependent CEA accessibility was not directly quantified, and off-lung CAR-Treg accumulation was observed.
Alcaraz-Serna et al., 2026 (35)	CAlleR-Treg	Bet v 1	Birch pollen-induced allergic airway inflammation in mice	*In vitro* + *in vivo* preclinical	Reduced eosinophilic inflammation, TH2 cytokines, mucus production, and AHR.	Activation depended on allergen presentation and noncompeting IgG, with limited support from the human serum samples examined.
Ward et al., 2018 (33)	CAR-T	mIgE	*In vitro*	*In vitro* proof-of-concept	Recognized mIgE-positive cells despite soluble IgE and did not activate IgE-loaded mast cells.	Efficacy, persistence, and safety were not evaluated in an asthma model.
Sun et al., 2026 (34)	CAR-T	FcϵRI	*In vitro* studies and an HDM-induced murine asthma model	Conference abstract; preliminary preclinical evidence	Reported activity against FcϵRIα-expressing targets *in vitro* and reductions in basophils and eosinophilic inflammation in an HDM-induced mouse model.	Methods and outcomes could not be independently assessed from the publicly available record.
Sun et al., 2025 (32)	CAR-T	IL-5R, IL-13R, FcϵRI	*In vitro*; murine asthma model	Conference abstract; preliminary preclinical evidence	IL-5-directed CAR-T was reported to produce greater reductions in eosinophilic infiltration and pulmonary inflammation than IL-13R- or FcϵRI-directed constructs in the tested murine model.	Comparative findings require confirmation in a peer-reviewed study with complete quantitative data.
Chen et al., 2022 (16)	CCAR-T	IL-5Rα	*In vitro* cell assays; HDM- and OVA-induced mouse models of allergic airway inflammation; preclinical study	*In vitro* + *in vivo* preclinical	Depleted IL-5Rα-positive eosinophils and progenitors, reduced airway inflammation, and provided protection for approximately 3 months.	Chronic therapeutic efficacy, dose-response relationships, long-term toxicity, and human applicability remain uncertain.
Jin et al., 2024 (15)	5TIF/5TIF4	IL-5Rα	Immunocompetent mouse models of asthma	*In vivo* preclinical	A single 5TIF4 infusion durably suppressed eosinophilia and type 2 airway inflammation without lymphodepletion.	The respective contributions of eosinophil depletion and IL-4 mutein secretion cannot be separated, and the long-term safety of gene editing remains unknown.
Mohammad Taheri et al., 2024 (23)	CAR-NK	Proposed asthma-associated immune targets	–	Review/conceptual; no direct asthma experimental validation	Proposed off-the-shelf manufacturing, native NK-cell recognition, and potentially lower toxicity.	Conceptual proposal without direct asthma-specific experimental validation.
Taheri et al., 2024 (22)	CAR-NKT	Proposed IgE-axis and immunoregulatory targets	–	Review/conceptual; no direct asthma experimental validation	Outlined potential CAR-NKT targeting and immunoregulatory strategies for asthma.	Conceptual proposal without direct asthma-specific experimental validation.

## Potential clinical toxicities and safety-mitigation strategies

Potential clinical toxicities of CAR-engineered cell therapy in asthma may arise from immune-effector-cell activation, preparative treatment, target biology, and product design (40, 41, 64). CRS and ICANS require particular attention because systemic cytokine release, hypoxemia, hemodynamic instability, and neurological dysfunction can become life-threatening (41, 65, 66). In severe asthma, respiratory manifestations of CRS may also be difficult to distinguish from an acute exacerbation, infection, or an infusion reaction (7, 41, 65). Other systemic complications may include immune effector cell-associated hemophagocytic lymphohistiocytosis-like syndrome (IEC-HS), prolonged cytopenia or immune effector cell-associated hematotoxicity (ICAHT), and infection (66–68). Additional product-related risks arise from excessive persistence, regulatory-cell instability, gene editing, and allogeneic cell platforms (37, 59, 64). Safety evaluation should therefore combine established immune-effector-cell monitoring and rescue frameworks with asthma-focused target validation, pulmonary monitoring, exposure control, reversibility, and long-term genetic surveillance (43, 64, 69). **Table 2** summarizes potential manifestations and mitigation strategies. The monitoring and mitigation approaches summarized here are extrapolated primarily from established CAR-T toxicity-management frameworks in oncology and autoimmune disease and have not been validated specifically for CAR-engineered cell therapy in asthma.

**Table 2:** Safety considerations and mitigation strategies for CAR-engineered cell therapy in asthma.

Safety concern	Potential implications in asthma	Monitoring and mitigation
CRS	Fever, hypotension, hypoxemia, capillary leak, and organ dysfunction may accompany systemic immune activation. Pulmonary involvement could aggravate airflow limitation and may resemble asthma exacerbation, infection, or an infusion reaction.	Baseline infection and lung-function assessment; close early monitoring; ASTCT grading; oxygen, vasopressor, and organ support when indicated; tocilizumab for clinically significant CRS; corticosteroids for severe, persistent, or refractory CRS; predefined criteria for intensive-care escalation.
ICANS	Impaired attention, aphasia, confusion, tremor, seizures, reduced consciousness, and rarely cerebral edema may occur with or after CRS. Hypoxemia, infection, metabolic abnormalities, stroke, medication effects, and seizures may complicate diagnosis.	Baseline neurological examination; serial ICE scoring; exclusion of alternative causes; corticosteroids as the principal anti-inflammatory treatment; seizure-directed therapy and intensive care when indicated. Tocilizumab should not be used as primary treatment for isolated ICANS.
IEC-HS, cytopenia, and infection	Hyperinflammation after CRS may present with rising ferritin, new or progressive cytopenias, coagulopathy, transaminitis, and multiorgan dysfunction. Cytopenia, conditioning, and immune-cell depletion may also predispose to infection, delayed immune recovery, and impaired immunoglobulin responses.	Serial blood counts, ferritin, liver-function and coagulation testing; prompt exclusion of infection; corticosteroids with or without anakinra under specialist guidance; transfusion or growth-factor support and antimicrobial prophylaxis when indicated.
Target-related immune and pulmonary toxicity	Recognition or depletion of IL-5Rα-, mIgE-, or FcϵRI-associated cells may cause prolonged eosinophil depletion, altered IgE-dependent immunity, mast-cell or basophil mediator release, bronchospasm, hypoxemia, or local pulmonary inflammation.	Human target-expression, antigen-density, and cross-reactivity studies; mast-cell and basophil activation assays; monitoring of eosinophils, immunoglobulins, infections, oxygenation, and lung function; cautious dose escalation and rapid interruption of persistent activity.
Product-design and long-term risks	Persistent, gene-edited, or integrating-vector products may cause prolonged immune perturbation, constitutive payload effects or delayed off-target toxicity. Allogeneic products may also undergo host rejection or, depending on product composition, cause GVHD.	Prefer controllable or depletable products, inducible payload expression, and minimal conditioning; perform genomic and product-purity testing; monitor CAR-cell persistence and clonality; incorporate a termination mechanism and conduct long-term clinical surveillance.

## Clinical translation and practical implementation

Most asthma-directed CAR studies remain confined to *in vitro* experiments and mouse models. Translation will therefore require a rigorous, staged preclinical validation framework (57, 58, 64). In addition to chronic OVA and HDM models, testing should include steroid-resistant disease, repeated allergen challenge, fixed airflow limitation, and fungal sensitization to evaluate target expression, CAR-cell trafficking, local effects, and injury to normal tissues across multiple disease contexts (57, 70, 71).

Patient stratification will also determine whether an acceptable risk-benefit ratio can be achieved (7, 64). Potential participants should first meet a strict definition of severe asthma (7, 72). A more appropriate population for early studies may comprise patients with recurrent severe exacerbations and long-term oral corticosteroid dependence who have an inadequate response to or cannot tolerate, otherwise appropriate targeted biologics (7, 11, 72). Selection should also be aligned with the mechanism of the specific CAR product (2, 64). FeNO, blood eosinophil counts, and total IgE may contribute to composite phenotyping, but none can substitute on its own for direct assessment of target expression and function (2, 73, 74). Given the absence of clinical safety or efficacy data, current evidence does not support clinical use of CAR-engineered cells in patients whose asthma is adequately controlled with established therapies.

Beyond biological efficacy and acute toxicity, practical implementation will depend on whether a reproducible cell product can be manufactured at an acceptable cost and within a clinically appropriate timeframe (75, 76). Autologous products may be limited by manufacturing delays, batch-to-batch variability, and patient fitness, whereas allogeneic or off-the-shelf products introduce additional risks of host rejection, graft-versus-host disease, and regulatory complexity (50, 64, 75). Clinical translation will require GMP-compliant production, validated release criteria for product identity, purity, potency, and sterility, and predefined procedures for manufacturing failure or out-of-specification products (75, 76).

## Knowledge gaps and future research priorities

Despite encouraging proof-of-concept findings, the development of CAR-based therapies for asthma remains at an early preclinical stage. Several knowledge gaps must be addressed before clinical translation. The distribution, density, and temporal stability of candidate surface antigens have not been fully characterized across airway and lung-resident cell populations, asthma endotypes, disease stages, and treatment backgrounds (2, 3). This uncertainty complicates the assessment of target coverage and on-target, off-tissue toxicity (39, 77). The level and duration of CAR-cell persistence required for sustained disease control also remain unclear (17, 40). Prolonged depletion or modulation of immune-cell subsets may affect immune homeostasis, tissue repair, and host defense (29, 41). In addition, current experimental models do not adequately reproduce the chronic and heterogeneous nature of human asthma (57). Validated biomarkers for patient selection remain lacking, and conditioning strategies and long-term safety-monitoring frameworks have not been established (7, 42, 64).

Several priorities should guide future work. First, maps linking targets, cell types, and disease endotypes should be generated from human asthma and normal tissues to validate candidate surface antigens and assess safety (39); IL-10 expression driven by the PDCD1 (PD-1) promoter could be evaluated as a CAR activation-responsive immunoregulatory module in CAR-Treg cells (78). Second, multi-target and secretory CARs should combine broader activity with safety switches, antigen-inducible expression, and drug-controlled systems (43, 56, 63). Third, humanized and ex vivo human models should be developed to capture chronic remodeling, steroid resistance, recurrent exacerbations, and complex comorbidity (58, 70). Fourth, patient-selection criteria should be matched to CAR mechanism and incorporate blood and airway eosinophils, FeNO, allergen sensitization, oral corticosteroid burden, and prior responses to biologics (2, 7, 73). Fifth, first-in-human trials should prioritize products that require no lymphodepletion or only low-intensity conditioning and that include mechanisms for termination or cell depletion (42, 43, 64). Low starting doses with stepwise escalation should be used, together with long-term surveillance for delayed toxicities, CAR-cell persistence, and changes in immune function (41, 79).

## Conclusion

The available preclinical studies provide proof-of-concept that CAR-engineered cells can target pathogenic immune populations and deliver antigen-dependent immunoregulatory functions in asthma (15, 16, 36). These findings support the biological feasibility and research value of CAR-based approaches, particularly as programmable platforms for investigating durable, target-directed, and multifunctional interventions in selected type 2-high asthma endotypes (15, 29). But major uncertainties concern the relevance of current models to human disease and whether durable control can be achieved without disrupting normal immune function. Translation will require a favorable risk-benefit balance in a chronic, nonmalignant disease for which effective treatments are already available (6, 40, 64). Future studies should therefore prioritize safety, controllability, and rigorous human target validation (39, 43). If clinical evaluation is pursued, initial studies should be restricted to carefully selected patients with severe asthma that remains uncontrolled despite established therapies (6, 7).
